# Foreseeable Co-occurring O_3_ and PM_2.5_ Pollution in Eastern China Driven by Climate Teleconnections

**DOI:** 10.1021/acsenvironau.5c00164

**Published:** 2025-10-07

**Authors:** Xiaorui Zhang, Meng Gao, Gregory R. Carmichael

**Affiliations:** † Key Laboratory for Geographical Process Analysis and Simulation of Hubei Province, College of Urban and Environmental Sciences, 624722 Central China Normal University, Wuhan 430000, China; ‡ Department of Geography, 26679 Hong Kong Baptist University, Hong Kong SAR 999077, China; § John A. Paulson School of Engineering and Applied Sciences, Harvard University, Cambridge, Massachusetts 02138, United States; ∥ Department of Chemical and Biochemical Engineering, The University of Iowa, Iowa City, Iowa 52242, United States

**Keywords:** surface ozone pollution, particulate matter pollution, air pollution complex, climate teleconnections, seasonal prediction

## Abstract

The co-occurrence of surface ozone (O_3_) and particulate matter (PM_2.5_) pollution (COP) has been frequently observed in China, particularly in the North China Plain (NCP) during warmer months, posing significant threats to human health and ecosystems. However, the impact of climate factors on COP remains inadequately understood. This study identifies three major modes of interannual variability in the COP frequency in Eastern China, revealing a consistent spatial pattern, a North–south dipole, and heightened sensitivity in coastal regions. These modes are linked to preseasonal cooling sea surface temperatures (SSTs) in the Western Pacific Ocean, Arctic sea ice (SI) loss near the Barents Sea, and North Atlantic tripole SST anomalies associated with the North Atlantic Oscillation, respectively. Both observations and model simulations confirm that Western Pacific cooling suppresses the Western Pacific subtropical high, promoting pollutant accumulation in the NCP; Barents Sea SI loss triggers atmospheric wave trains, facilitating water vapor transport to southern China and air pollutants transport to Northern China, resulting in a North–south dipole in COP frequency; and North Atlantic Oscillation (NAO)-driven SST anomalies generate westerly wind anomalies, driving pollutants to coastal regions of Eastern China. A model that incorporates preseasonal SST and SI signals is demonstrated to be capable of predicting COP frequency three months in advance. Our results could allow the Chinese government to improve plans for pollution control and safeguard the health of both humans and ecosystems.

## Graphical abstract

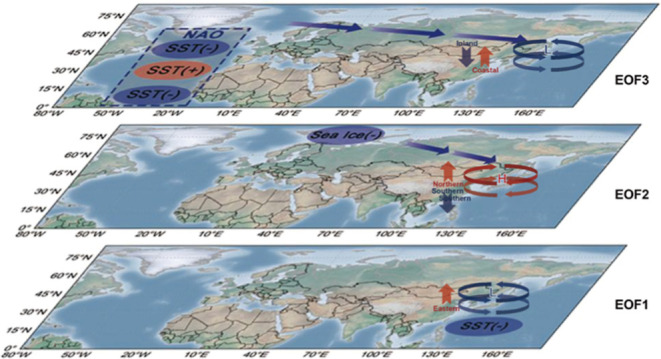

## 1 Introduction

Over recent decades, rapid industrialization, accelerated urbanization, and soaring energy consumption have resulted in severe air pollution across China.^1–3^ This air pollution complex is mainly characterized by elevated concentrations of ozone (O_3_) and fine particulate matter (PM_2.5_), drawing growing concern among the public and policymakers.^3^ Although the implementation of stringent clean air policies has led to a decline in PM_2.5_ concentrations since 2013, PM_2.5_ pollution episodes continue to occur frequently in China’s major city clusters.^2,4,5^ Meanwhile, reductions in nitrogen oxide (NO_*x*_) emissions have unintentionally contributed to rising levels of O_3_ under the volatile organic compound (VOC)-limited regimes prevailing in many urban areas.^6^ Declining PM_2.5_ has been linked to increased O_3_ concentrations by slowing the removal of hydroperoxyl radicals, weakening aerosol–radiation interaction.^6–8^ As a result, co-occurrence of surface O_3_ and PM_2.5_ pollution (COP) has been commonly observed in China during warmer months.^9–11^ Exposure to either PM_2.5_ or O_3_ is associated with an elevated risk of mortality, respiratory diseases, etc., while synergistic effects have been reported for exposure to COP.^12–15^ Additionally, the adverse impacts of COP on net primary productivity were also found in China.^16^

The formation of COP reflects the combined influence of emissions and meteorological conditions.^9,17,18^ Meteorological drivers become particularly dominant during extreme pollution events, contributing over 50% to haze and 70% to photochemical pollution episodes.^19–21^ COP events, featuring co-occurring extreme levels of both O_3_ and PM_2.5_, are thus significantly modulated by meteorological conditions.^9,22^ However, previous studies have mainly focused on local meteorological factors or synoptic-scale systems. Emerging evidence links the meteorological conditions conducive to O_3_ or PM_2.5_ pollution individually to large-scale climate drivers such as sea surface temperature (SST) anomalies and Arctic sea ice (SI) variability.^23–28^ For instance, Ma and Yin^24^ demonstrated that Arctic SI loss is associated with enhanced summer O_3_ pollution in Eastern China by a Eurasia-like Rossby wave train, with an 83% likelihood of O_3_ episodes under strong SI anomalies. Similarly, Arctic SI loss has been tied to increased winter PM_2.5_ levels in Eastern China by intensifying the Siberian High and strengthening northerly winds over the North China Plain (NCP), contributing to ∼50% of PM_2.5_ variability during anomalous years.^29^ Given the amplified damage from compound extremes,^14,30^ it is imperative to better understand COP at larger scales, particularly regarding its predictability. Yet, how climate factors modulate the interannual variability of COP remains largely unexplored. Building on our earlier work linking large-scale climate drivers to wintertime aerosol pollution in India,^31^ and co-occurrence of heatwave and O_3_ pollution in China,^32^ we hypothesize that preseasonal climate signals can provide predictive insights into COP frequency in China. Such forecasts could enable more proactive mitigation strategies, reducing the compounded impacts of COP on public health and the ecosystem.

This study aims to clarify the primary climate patterns driving the interannual variability of COP in China, where a significant portion of the world’s population is exposed to the dual burden of these pollutants. Using reconstructed long-term records of the concentrations of O_3_ and PM_2.5_ in China, we applied Empirical orthogonal function (EOF) analysis to decompose detrended COP frequency. Our results reveal that these modes are strongly modulated by meteorological conditions through teleconnections involving SST and SI anomalies. Both statistical analyses and Community Earth System Model version 2.1.3 (CESM version 2.1.3) experiments were employed to elucidate the mechanisms linking SST and SI variability to COP patterns. Based on these findings, we developed a regression model that enables seasonal prediction of COP frequency up to three months in advance, offering a tool for improving early warning systems and informing targeted pollution mitigation strategies.

## 2 Materials and Methods

### 2.1 Ground-Level O_3_ and PM_2.5_ Data

Satellite derived daily ground-level concentrations of O_3_ and PM_2.5_ in China. Daily MDA8 O_3_ data with a horizontal resolution of 0.1° × 0.1° in China were obtained from Gao et al.^32^ This data set was reconstructed using the extreme gradient boosting algorithm, incorporating inputs such as anthropogenic emissions, meteorological variables, land covers, etc., which was also used in our previous explorations.^32–34^ Daily PM_2.5_ concentrations were taken from the Long-term Gap-free High-resolution Air Pollutants concentration data set (LGHAP).^35^ The LGHAP v2 PM_2.5_ data during 2005–2021 at a 1 km horizontal resolution were derived through an integration of satellite retrievals, ground-based observations, and numerical simulations based on machine learning methods. Due to different horizontal resolutions, PM_2.5_ data were bilinearly interpolated onto grids of 0.1° × 0.1°. Quality control during satellite retrieval included the removal of outliers and unification of time scales to daily average for PM_2.5_ and daily maximum 8 h average for O_3_.^32,35^ The reliability of both data sets has been validated against ground-based observations from the China National Environmental Monitoring Center (CNEMC). For O_3_ and PM_2.5_, the respective correlation coefficients reach 0.94 and 0.95, while the root-mean-square errors (RMSEs) are 14.67 μg m^–3^ and 12.03 μg m^–3^, indicating high accuracy and suitability for long-term spatial–temporal analysis.^32,35^

As suggested previously that COP displays relatively higher frequency during May, June, and July,^9,18^ this study focused on variations of COP during May to July over 2005–2021. To eliminate the influence of changing anthropogenic emissions, we defined COP days as those when MDA8 O_3_ and daily averaged concentrations of PM_2.5_ both exceeded the 95th percentile of their respective values. The values were determined for May–July of all the grids of Eastern China (17.5–50°N, 98–125°E) with a three-year moving window, as illustrated in Figure 1. The spatial distribution pattern of detrended COP closely resembled that of the COP defined by the grade II National Ambient Air Quality Standard (NAAQS) of China (GB3095-2012) (Figure S1A,B). The variations of threshold values for PM_2.5_ and O_3_ were consistent with the contribution of anthropogenic emissions to air pollutants.^2,6^ Thus, the trending method in this study can mostly eliminate the influence of changing anthropogenic emissions. As shown in Figures S1 and S2, a sensitivity analysis was conducted to assess the influence of the chosen percentile threshold and window length on the COP definition. Alternative thresholds (90th and 80th percentiles) were tested and found to produce lower concentration thresholds, which led to a substantial overestimation of COP frequency by classifying moderate pollution days as extreme events (Figure S1F,G). The 95th percentile was retained as it yields thresholds that align closely with China’s grade II NAAQS, ensuring a focus on scientifically and policy-relevant extreme events. The sensitivity to window length was also evaluated (Figure S2). A 1 year window was found to remove both emission trends and meteorologically driven interannual variability, the latter of which is a key target of our investigation. While 3 year and 5 year windows produced similar detrending effects, a 3 year window was selected to minimize edge effects in our 17 year time series, ensuring threshold comparability across most years.

**Figure 1:**
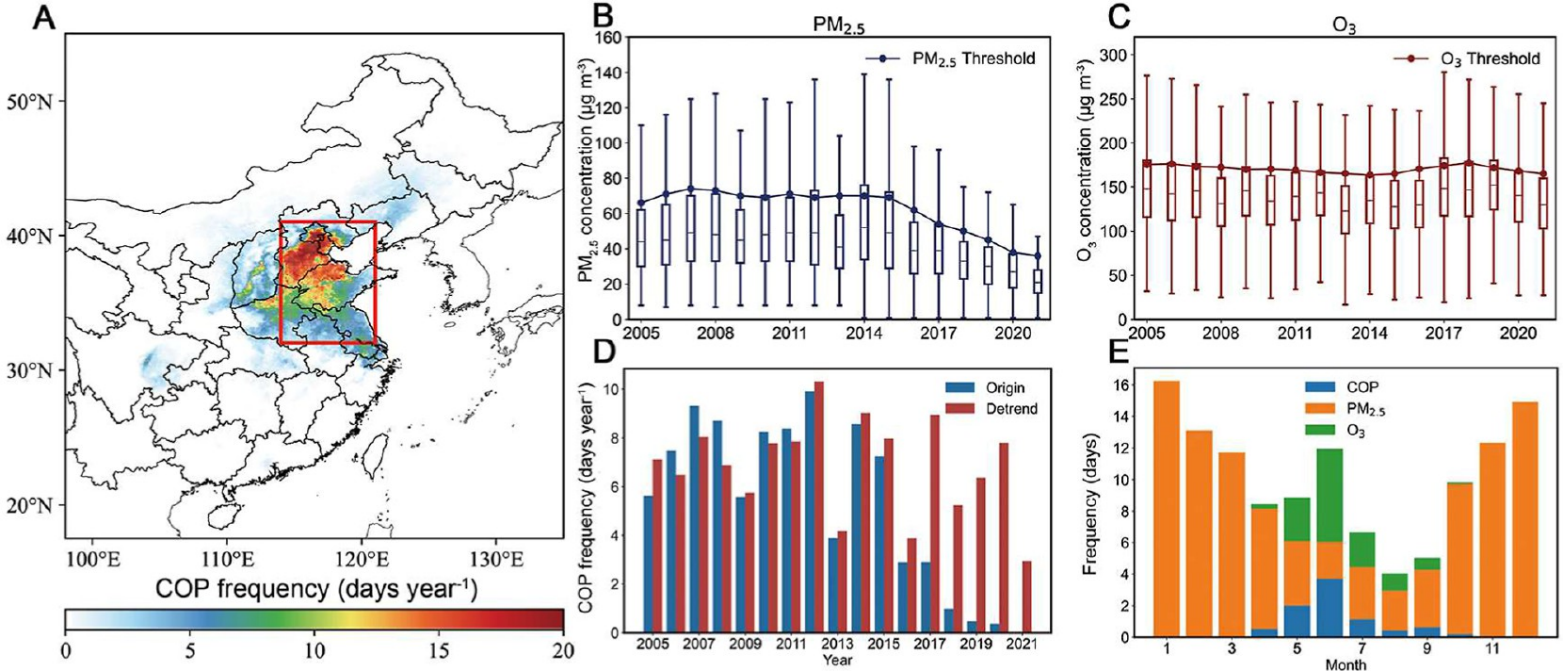
Spatial distribution and temporal variations of COP frequency. (A) Spatial distribution of averaged detrended COP frequency (days year^–1^) during May–July from 2005 to 2021 in Eastern China (red rectangle denotes areas of the NCP). Interannual variations of (B) PM_2.5_ (μg m^–3^) and (C) O_3_ concentrations (μg m^–3^) (box plots) in Eastern China, and their associated threshold values (Line). (D) Interannual variations of original (blue bars) and detrended (red bars) COP frequency (days year^–1^) in NCP. (E) Averaged frequency (days) of COP (blue bars), only PM_2.5_ pollution event (orange bars) and only O_3_ pollution event (green bars) in each month during 2005–2021.

### 2.2 Meteorological Reanalysis

Meteorological variables including SST, geopotential height, zonal and meridional winds, relative humidity, precipitation, and surface direct short-wave radiation flux at a spatial resolution of 0.25° × 0.25° were obtained from the European Centre for Medium-Range Weather Forecasts (ECMWF) ERA5 data set,^36^ which are highly related to COP events. Met Office Hadley Centre offers the monthly SI concentration data at a horizontal resolution of 1.0° × 1.0°.^37^ The North Atlantic Ocean (NAO) index was taken from the NOAA Climate Prediction Center (CPC).

### 2.3 Statistical Methods and Wave Activity Analysis

The interannual spatiotemporal variations of detrended COP from 2005 to 2020 in China were decomposed by EOF analysis. The first three modes were mainly focused, which were significantly separated from other modes.^38^ To investigate the propagation of atmospheric Rossby waves, the horizontal wave activity flux (WAF), obtained from the conservation of wave-activity momentum, was calculated:^39^

1 $$W=\frac{1}{2\left|\mathrm{U̅}\right|}\left[\begin{array}{l} \mathrm{u̅}\left({\Psi_{x}^{‘}}^{2}-\Psi^{\prime }\Psi_{xx}^{‘}\right)+\mathrm{v̅}\left(\Psi_{x}^{‘}\Psi_{y}^{‘}-\Psi^{\prime }\Psi_{xy}^{‘}\right)\\ \mathrm{u̅}\left(\Psi_{x}^{‘}\Psi_{y}^{‘}-\Psi^{\prime }\Psi_{xy}^{‘}\right)+\mathrm{v̅}\left({\Psi_{y}^{‘}}^{2}-\Psi^{\prime }\Psi_{yy}^{‘}\right) \end{array}\right]$$

where ψ represents the geostrophic stream function, with subscripts indicating partial derivatives. *U* refers to the horizontal wind velocity, while *u* and *v* denote the zonal and meridional wind components, respectively. Additionally, W signifies the 2D Rossby wave activity flux (WAF).

To predict COP in China, a multivariable linear regression model was developed using anomalies of SST and SI as predictors. To assess the performance of various combinations of model predictors, we calculated AIC values.

### 2.4 CESM Model Experiments

The CESM v2.1.3 was utilized to investigate the responses of COP frequency to SST and SI anomalies. The horizontal resolution and vertical layers of CESM were set to be 0.94° × 1.25° and 70, respectively,^40^ and the FWHIST component was configured. The atmospheric components were derived from the Community Atmosphere Model version 6, and chemical and land processes were represented using the Whole Atmosphere Community Climate Model version 6.^41^ Biogenic emissions were calculated online through the Model of Emissions of Gases and Aerosols from Nature (MEGAN) version 2.1, integrated within the CLM5 model.^42^ SST and sea ice concentrations were prescribed in the experiments, which allowed SST and sea ice anomalies to be imposed for single forcing experiments. A control case was forced with SST data from monthly varying climatology (CESM_ctl_). Corresponding with the decomposed climate modes, three sensitivity cases were conducted by imposing SST anomalies associated with the western Pacific SST index (SST_wp_) (CESM_wp_) and NAO index (CESM_NAO_) and SI anomalies associated with the Barents SI area index (SIAI) index (CESM_SI_), as shown in Figure S3. All experiments were run from January to August 2010. Given the biases of simulation,^32,33^ simulated results were employed to assess the direction of the response rather than exact magnitudes.

## 3 Results

### 3.1 Spatial Distribution and Interannual Variability of COP Frequency

COP events are most frequent in the NCP during May–July, with mean occurrences exceeding 8 days year^–1^ (Figure 1). A COP day was defined when daily concentrations of both pollutants exceed the threshold value, specifically the 95th percentile of pollutant concentration during May–July over moving windows of three consecutive years in Eastern China. The average threshold concentrations for PM_2.5_ and O_3_ were estimated to be 70.4 and 170.9 μg m^–3^, respectively, over 2005–2012, closely aligning with China’s grade II NAAQS of 75 μg m^–3^ for daily PM_2.5_ and 160 μg m^–3^ for daily maximum 8 h average O_3_. Following the implementation of stringent clean air policies,^2,4^ PM_2.5_ concentrations in Eastern China declined substantially since 2013, resulting in a downward trend in PM_2.5_ threshold values (Figure 1B). This adaptive thresholding helps minimize the confounding influence of shifting anthropogenic emissions on the COP frequency. In contrast, the O_3_ thresholds increased from 2013 to 2016, followed by a decline after 2017 (Figure 1C). It is consistent with previous studies that anthropogenic emissions exert negative contribution to O_3_ over 2013–2016 but positive over 2017–2019.^43^ Therefore, interannual variations of detrended COP frequency are more consistent with the original frequency observed before 2013, but different thereafter (Figure 1D). The distributions of COP defined by lower percentiles are similar to those of NAAQS but show obviously higher frequency (Figure S1).

The spatiotemporal correlation between O_3_ and PM_2.5_ reveals a complex pattern across Eastern China (Figure S4A). A significant positive correlation (*r* >0.4, *p* <0.05) is prevalent in southern China, indicating that pollutants tend to covary under similar meteorological conditions. In contrast, a negative correlation is observed over the Taklimakan and Gobi deserts due to the scavenging effect of dust on O_3_, which also impacts downstream areas. In the NCP, the positive correlation between O_3_ and PM_2.5_ is relatively weak (*r* <0.25), primarily because high PM_2.5_ concentrations reduce O_3_ levels through the removal of hydroperoxyl radicals and aerosol–radiation interactions.^6–8^ Consequently, while a higher frequency of pollution events increases the likelihood of COP occurrences, elevated concentrations of individual pollutants do not necessarily promote COP events (Figure S4). The proportion of COP events is approximately 50% in NCP among the O_3_ pollution events and PM_2.5_ pollution events, respectively.

### 3.2 Major Modes of COP Frequency

Figure 2 illustrates the first three EOF modes of the detrended COP frequency in Eastern China. These modes, which account for 32%, 13%, and 11% of the total variance, are statistically distinguished based on the North Test.^38^ The first EOF (EOF1) pattern closely resembles the distributions of COP frequencies depicted in Figure 1A, reflecting a consistent spatial response across Eastern China. Its associated normalized principal component (PC1) exhibits significant interannual variability, peaking in 2012 and reaching a minimum in 2021. PC1 is strongly correlated with detrended COP frequency in NCP (*r* = 0.89, *P* <0.01). The second EOF (EOF2) reveals a North–south dipole pattern of COP in Eastern China, highlighting out-of-phase variations between the Yangtze River Delta (YRD) and Northern China. EOF3 presents a positive sensitivity in coastal areas of Eastern China, extending from Northeastern China to coastal regions of YRD. The values of PC3 are generally negative before 2016 and become positive thereafter.

**Figure 2:**
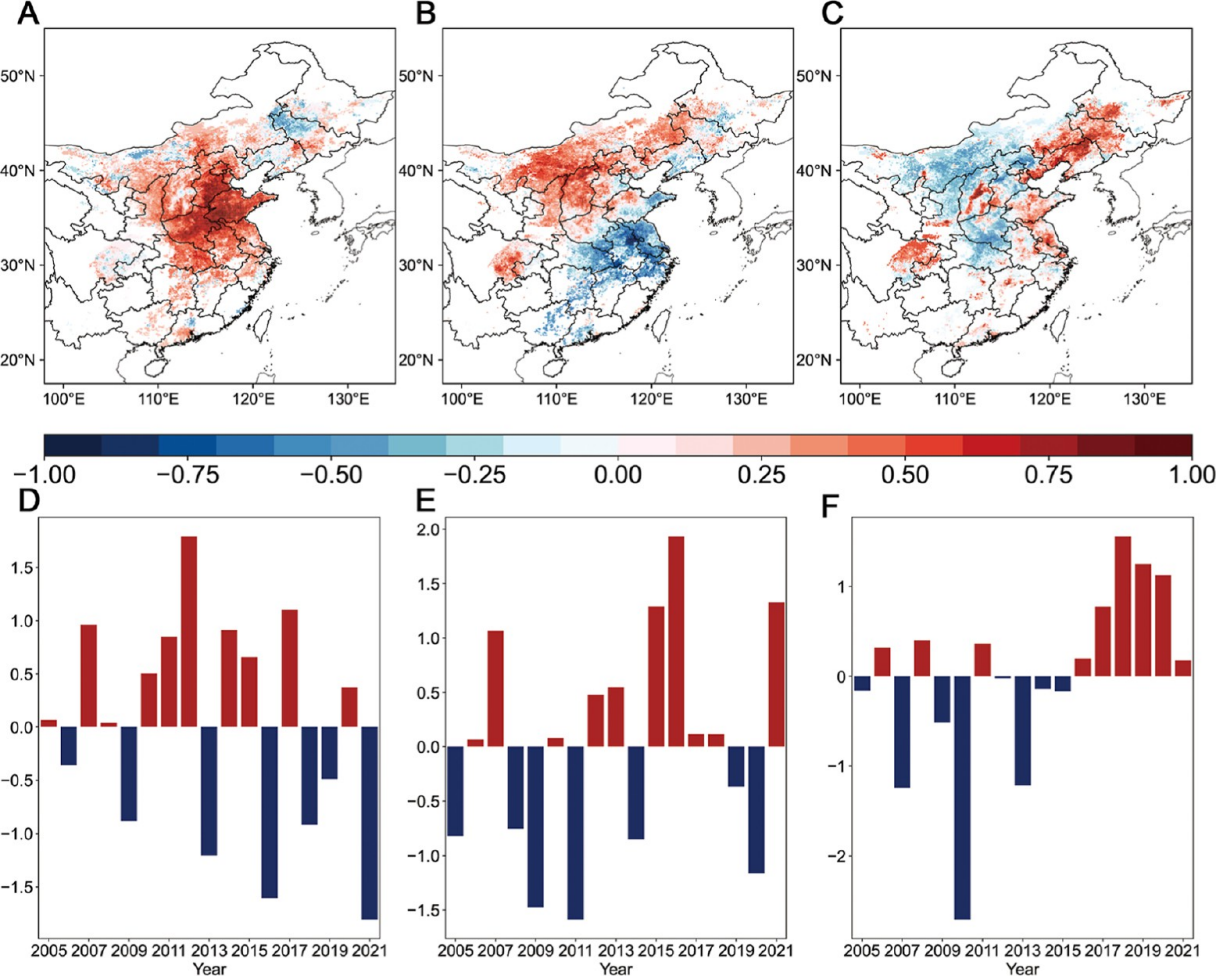
Features of the first three leading modes. Spatial patterns of (A) EOF1, (B) EOF2, and (C) EOF3. Interannual variations of the COP frequency of (D) PC1, (E) PC2, and (F) PC3 during May–July from 2005 to 2021.

To explore the climate patterns associated with the primary modes of COP frequency, correlation analyses were conducted between each principal component and the SST SI (Figure 3). We focused on February–April SST and April SI, which may provide insights for seasonal predictions of COP frequency during May–July. The monthly variation in Arctic SI generally shows an expansion until April, followed by ablation. The SI in April demonstrates significant persistence into subsequent months.^44,45^ Consequently, we selected April SI rather than the average SI from February to April. As illustrated in Figure 3A, PC1 exhibits a statistically significant negative correlation with the SST during February–April over the western Pacific Ocean region. Previous studies pointed out that western Pacific SST anomalies influence the evolution of East Asian summer monsoon,^46^ strength of Western Pacific Subtropical High (WPSH),^47^ and co-occurrence of heat wave and O_3_,^32^ which may have potential impacts on COP. PC2 is not highly correlated with SST (Figure 3C), but it displays a robust negative association with SI near the Barents Sea (Figure 3D). Previous studies have linked Barents Sea SI loss to precipitation anomalies,^25^ extreme drought events,^48^ and dust weather events in China,^49^ indicating its broader influence on midlatitude atmospheric circulation. Significant correlations between PC3 and SST are found in the North Atlantic region, presenting a tripole pattern (Figure 3E) and persisting to midsummer (Figure S2F). Such North Atlantic tripole SST anomalies during February–April are linked to NAO, with an intensified connection since the late 1980s.^50^ The lagged influence of spring NAO on summer climate over East Asia is well established and is believed to be modulated by the maintenance of the North Atlantic tripole SST pattern.^51,52^

**Figure 3:**
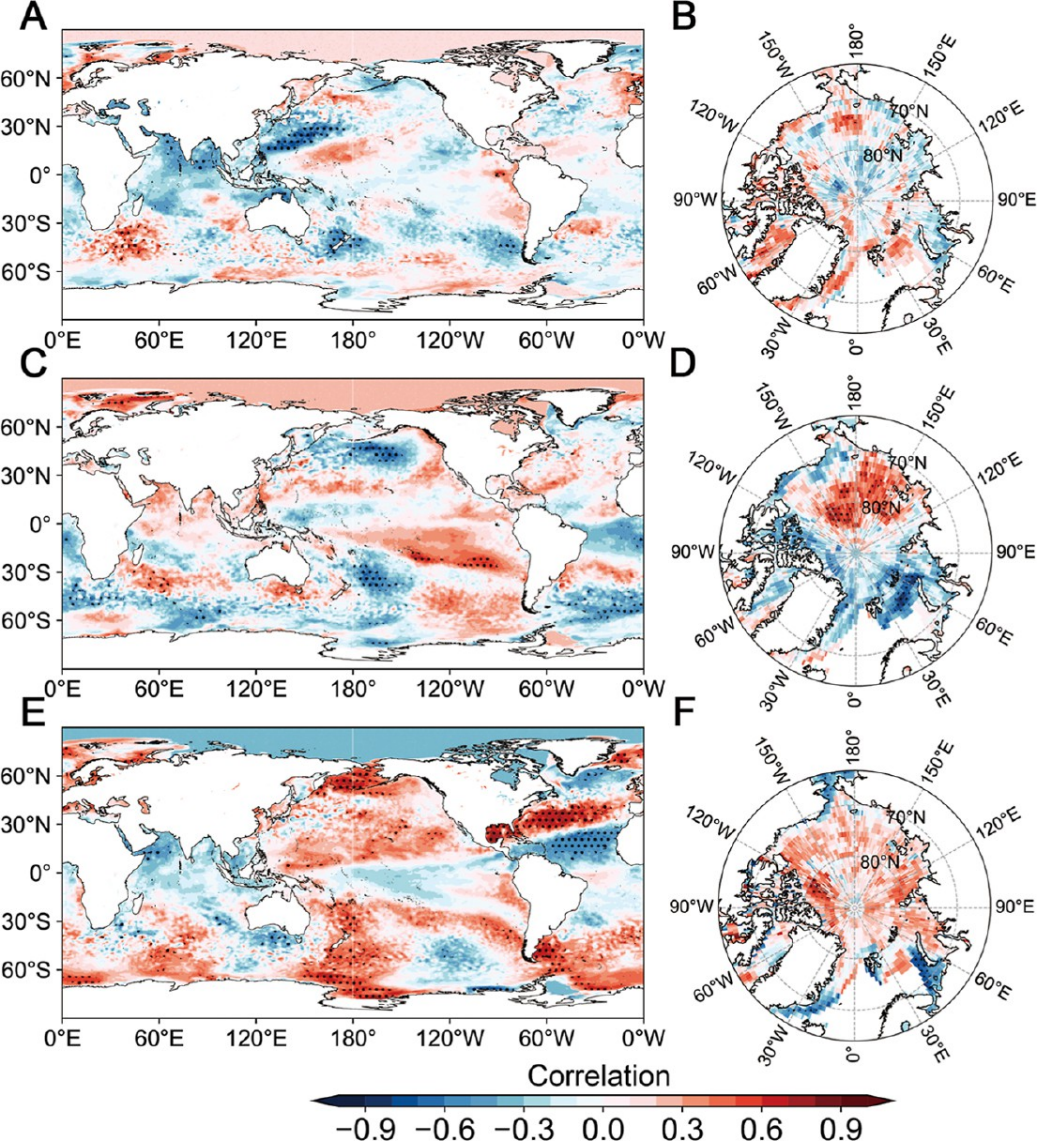
Correlations between the leading modes and SST/SI. Correlations between the first three modes and (A,C,E) SST during February–April. (B,D,F) SI during April from 2005 to 2021. Black dots denote areas with significant correlation (*P* <0.05).

### 3.3 Impacts of Cooling in the Western Pacific Ocean

As noted above, PC1 displays a significant negative correlation with February–April SST in the Western Pacific Ocean. The SST_wp_ is defined as the averaged SST during February–April over Western Pacific (10°N to 30°N, 130°E to 160°E), multiplied by −1. The SST_wp_ index is closely correlated with PC1 (*r* = 0.71, *P* <0.01), and the association persists into the subsequent May–July period, albeit with weaker intensity (*r* = 0.56, *P* <0.05) (Figure S5B). Persistent cold western Pacific SST anomalies suppress local convective activity, leading to thermodynamically unfavorable conditions for the development of the WPSH.^32,46^ Lou et al.^53^ further demonstrated that western Pacific SST anomalies can excite meridional Rossby wave trains propagating eastward to high latitudes, modulating circulation anomalies over the Northern Hemisphere extratropics. To verify the role of cooling SST over the western Pacific Ocean, the SST anomalies associated with the SST_wp_ index were imposed in the simulation. Figure S6 shows the differences between CESM_wp_ and CESM_ctl_ cases, which are considered as the influence of cooling in the Western Pacific Ocean. Western Pacific cooling during February–April suppresses the WPSH from May to July (Figures 4A and S6A). The induced anomalous cyclonic circulation over the Western Pacific enhances northerly winds across northern China, promoting the accumulation of air pollutants while simultaneously weakening moisture transport to the NCP (Figure S6B). Consequently, the NCP experiences reduced precipitation (Figure 4B), leading to less wet scavenging of PM_2.5_ and inhibiting heterogeneous reactions and photolysis of O_3_.^54–56^ Additionally, the reduced precipitation and clouds also allow more solar radiation flux on the ground (Figure 4C), which increases the surface temperature (Figure S6E). Higher solar radiation and temperature in NCP promote the development of planetary boundary layer height (PBLH) (Figure S6G), enhancing the vertical mixing and transport of O_3_ as well as its precursors. The production of O_3_ in NCP is accelerated by intensified photochemical reaction and enhanced emissions of VOCs under warmer conditions (Figure S6F).

**Figure 4:**
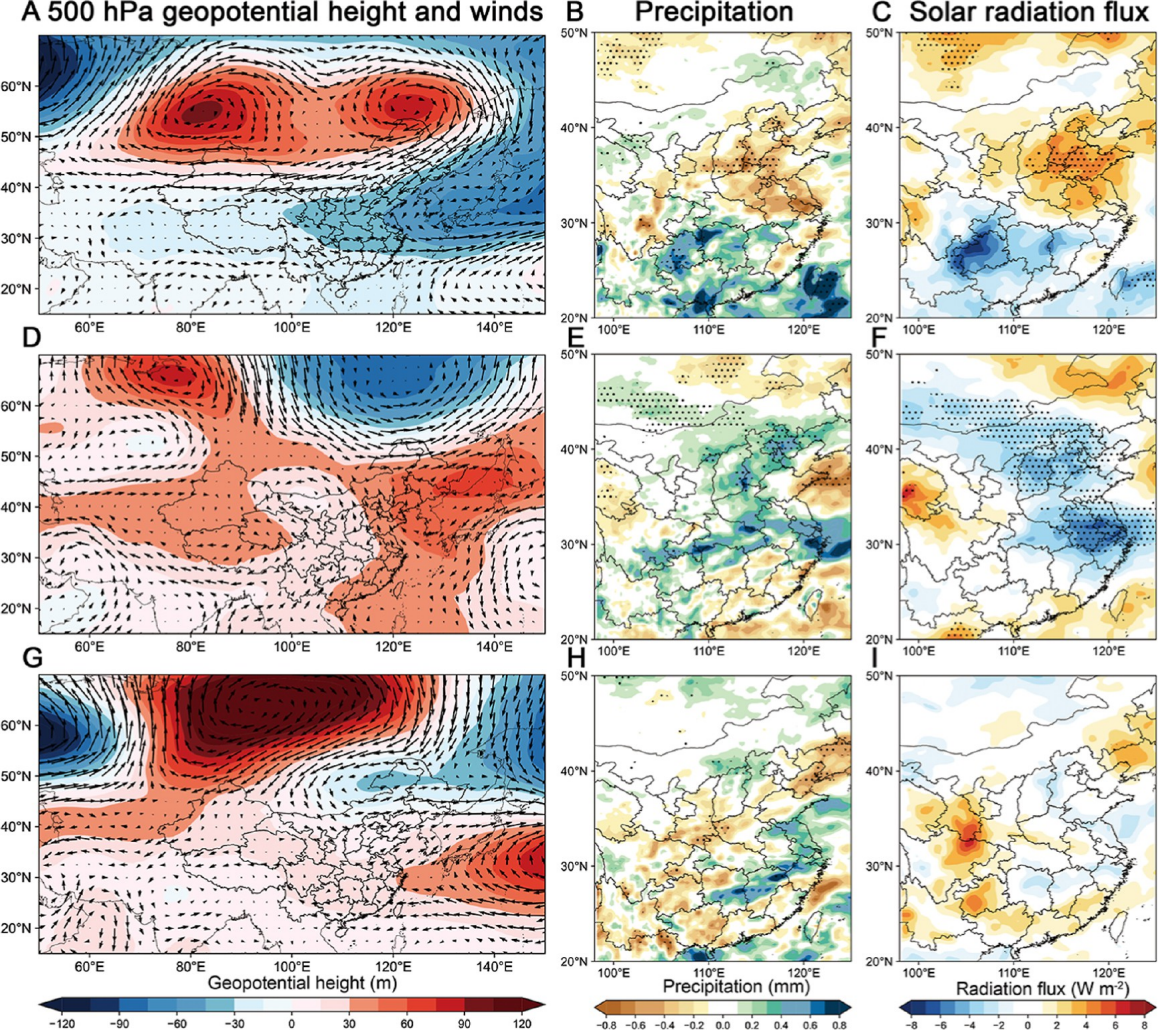
Meteorological patterns for leading modes. Regression of geopotential height (m) and winds (m s^–1^) at 500 hPa, total precipitation (mm) and solar radiation flux (W m^–2^) during May–July from 2005 to 2021 on (A,B,C) PC1, (D,E,F) PC2, and (G,H,I) PC3.

In response, simulated concentrations of both O_3_ and PM_2.5_ increase across the NCP (Figure 5A and D). The relative humidity and precipitation are relatively higher in the southern part of Eastern China, where the concentration of O_3_ and PM_2.5_ is slightly reduced. The occurrences of COP are not frequent in southern China (Figure 1A), where the reduction of air pollutants has a minimal impact on COP. Subsequently, cooling SST in the Western Pacific Ocean predominantly increases COP frequency over the NCP (Figure 5G), consistent with the spatial pattern of EOF1, reinforcing the critical role of western Pacific SST anomalies in driving interannual COP variability in Eastern China.

**Figure 5:**
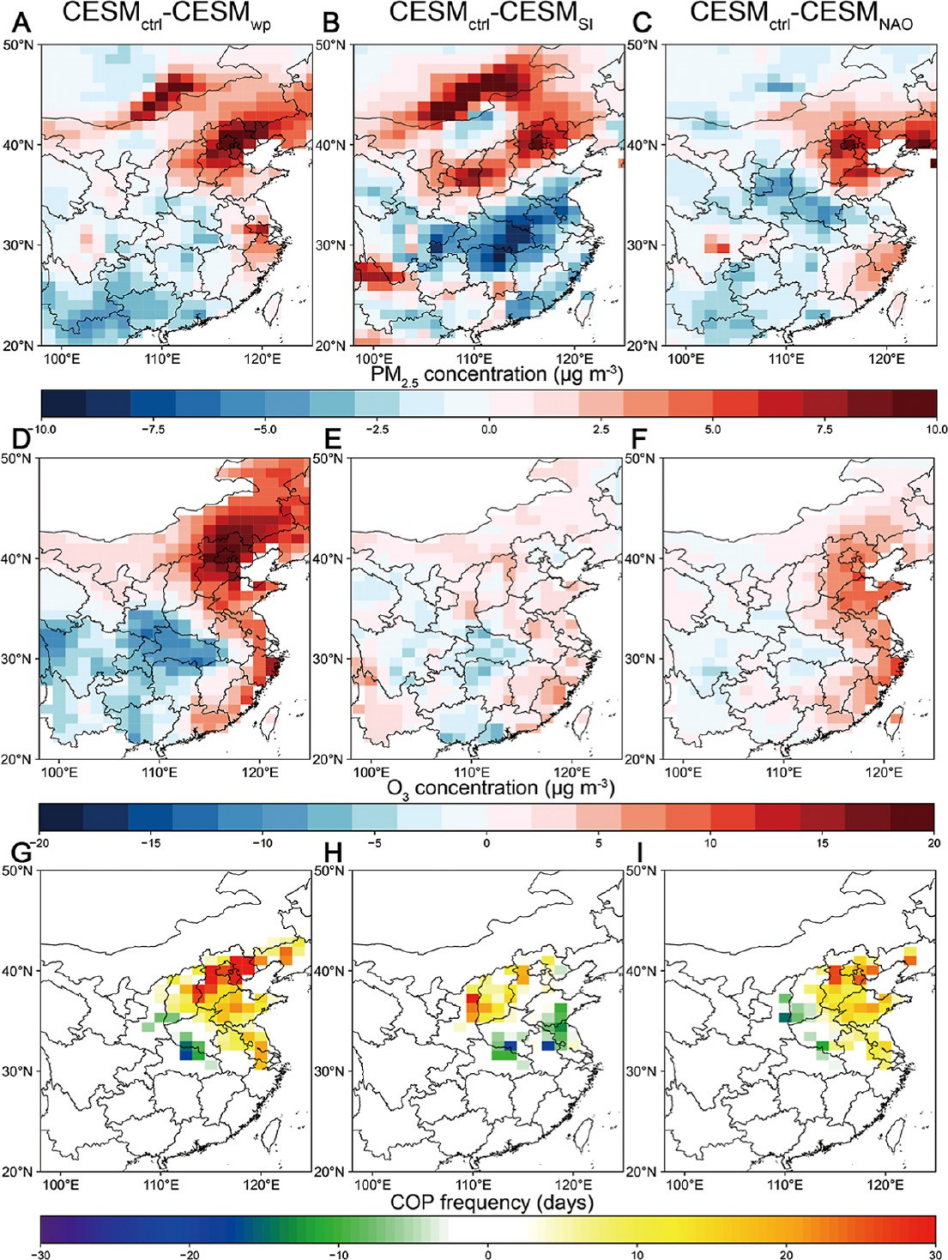
CESM-simulated responses of air pollutant concentrations and COP. CESM-simulated responses of (A,B,C) PM_2.5_ concentration (μg m^–3^), (D,E,F) O_3_ concentration (μg m^–3^), and (G,H,I) COP frequency (days) during May–July to SST_wp_, SIAI, and NAO.

### 3.4 Impacts of Arctic Sea Ice Loss near the Barents Sea

Barents Sea witnesses a pronounced negative relation between the SI area and PC2. Such negative relationship has a good seasonal persistence from April to midsummer (Figure S7A), consistent with documented memory effects of SI anomalies.^44,45^ Spring-to-summer SI loss near the Barents Sea enhances ocean–atmosphere heat exchange,^45^ leading to regional surface warming (Figure S7B) and elevated SST from May to July (Figure S5D). These thermal anomalies would act as a source of baroclinic disturbances, initiating large-scale atmospheric responses.^48^

To quantify this link, we define the SIAI as the negative area average of the SI area during April over 75°–78°N, 30°–60°E. The SIAI exhibits a significant positive correlation with PC2 (*r* = 0.68, *p* <0.01). To assess the causal impact of sea ice reduction, we performed a targeted CESM experiment (CESM_SI_) in which SI was reduced in the Barents Sea region. As demonstrated in Figure S8, differences between CESM_SI_ and CESM_ctl_ cases are regarded as the impacts of Arctic SI loss near the Barents Sea. Both ERA5 reanalysis and CESM simulations reveal an ice-loss-triggered Rossby wave train propagating from the Barents Sea to Eastern China (Figures S7C and S8A). This induces an anticyclonic anomaly over Northeast China, enhancing southeasterly flow across eastern China (Figure 4D), which promotes transport of water vapor to the YRD. Meanwhile, reduced solar radiation flux correlates with increased precipitation (Figure 4E and F), ultimately suppressing COP frequency in the YRD. Elevated PBLH is found in Shandong (Figure S8G), increasing surface O_3_ concentrations while reducing PM_2.5_ concentration by enhanced dispersion. Additionally, anomalous southeasterly winds redistribute air pollutants northward, decreasing the PM_2.5_ concentration in the YRD while increasing them in northern China. This contrasting response produces a meridional dipole in COP frequency across Eastern China, with decreased frequency in the south and increased frequency in the north (Figure 5H), in accordance with the North–south dipole pattern in EOF2.

### 3.5 Impacts of North Atlantic Tripole SST Associated with North Atlantic Oscillation

The interannual variability of COP frequency is also related to NAO, as evidenced by robust correlations between the PC3 and North Atlantic tripole SST anomalies persisting from February into midsummer (Figure S5F). Regressions of February–April 500 hPa geopotential height onto PC3 reveal a classic NAO structure, with negative anomalies over Iceland and positive anomalies over Azores (Figure S9). The NAO modulates Rossby wave propagation and thereby affects broader atmospheric circulation. During its positive phase, the NAO induces a cooling effect in the northern tropical Atlantic, triggering an anticyclonic response over the subtropical western North Atlantic^50^ and further inducing NAO-like dipole anomalies over the North Atlantic (Figure S9). Furthermore, the NAO often aligns with broader teleconnection patterns, such as the East Atlantic/West Russia (EA/WR) pattern.^57^ Originating in the North Atlantic, the EA/WR extends eastward across Europe and European Russia, exerting substantial influence on East Asian temperature and precipitation.^58^

The North Atlantic tripole SST anomalies associated with the NAO index were imposed in the CESM_NAO_ case. The wave activity flux and geopotential height responses in Figure S10 reveal that such SST anomalies trigger downstream development of subpolar teleconnections across northern Eurasia, generating negative geopotential anomalies over the Ural Mountain and Okhotsk Sea (Figures S10 and S11A). The resulting anomalous cyclonic circulation over the Okhotsk Sea leads to westerly wind anomalies across Eastern China (Figure 4G), enhancing the eastward advection of air pollutants from inland areas toward coastal regions. The atmospheric circulation anomalies induce modest meteorological changes, slightly reducing moisture transport and modestly elevating solar radiation along coastal regions (Figure 4H and I). The responses of temperature and VOCs emissions are weak in China to NAO (Figure S11). Higher O_3_ and PM_2.5_ concentrations in coastal regions are mainly attributed to westerly wind anomalies (Figure 5C and F). Accordingly, the responses of COP to NAO are obviously positive in coastal regions and slightly reduced in inland areas (Figure 5I), in agreement with the spatial signature of EOF3.

### 3.6 Statistical Seasonal Prediction

This study demonstrates that SST and SI anomalies exhibit good seasonal persistence from early spring to midsummer, exerting lagged influences on meteorological conditions associated with COP in Eastern China. These climate anomalies serve as effective modulators of interannual COP variability over the Eastern China during May–July. Based on these findings, the standardized SST_wp_ and NAO indices during February to April, along with the SIAI index during April, are selected as dominant predictors for COP frequency during May to July for the period of 2005–2021. Although the lagged influence of spring NAO on East Asian summer climate is noted in this study and previous studies,^52^ atmospheric patterns typically exhibit limited persistence. Consequently, the persistent North Tropical Atlantic SST (SST_NTA_, defined as the average SST from February to April over 30°N–40°N, 30°W–60°W), as documented by Luo et al.,^59^ serves as a complementary predictor to enhance the robustness of the predictive model. A seasonal prediction model is developed using multiple linear regression (MLR), represented by the following form:

2 $${COP=a}_{0}{+a}_{1}{SST}_{wp}{+a}_{2}{SIAI+a}_{3}{NAO+a}_{4}{SST}_{NTA}$$

where *a*_0_, *a*_1_, *a*_2_, *a*_3_, and *a*_4_ represent coefficients determined through the multivariable regression procedure. Seven combinations of three predictors were considered in the MLR model and evaluated using the Akaike information criterion (AIC). The model incorporating all the three predictors has the best performance with the smallest AIC of 62.36 in NCP (Table S1). The MLR model demonstrates its capability to predict the COP frequency three months ahead, achieving a correlation coefficient of 0.86 (*P* <0.01) over NCP (Figure 6). Furthermore, the inferred COP derived from 10-fold cross-validation also captures the variation of observed COP with a correlation coefficient of 0.71 (*P* <0.05), confirming the robustness of our regression model. The model also shows promising performance in COP prediction in Eastern China and YRD with a correlation coefficient of 0.68 (*P* <0.01) and 0.51 (*P* <0.05), respectively (Figure S12). Hierarchical partitioning of explained variance further reveals distinct regional patterns in the relative contributions of these climate drivers (Table S2). The model explains 74% of the total variance in the COP frequency in the NCP, where SST_wp_ dominates COP variability with a relative contribution of 61.1%, followed by SIAI (20.9%), SST_NTA_ (11.9%), and NAO (6.1%). Similarly, for Eastern China as a whole, the model accounts for 47% of the variance, with SSTwp remaining the primary driver (64.8% contribution), while SIAI (21.7%), SST_NTA_ (7.5%), and NAO (5.9%) play secondary roles. In contrast, the YRD exhibits a more balanced influence pattern: SIAI (29.6%) and SST_wp_ (36.8%) emerge as codominant predictors, with NAO (15.4%) and SST_NTA_ (18.2%) also exerting non-negligible influences. The variance decomposition results are consistent with the spatial patterns of the EOF modes. SST_wp_ emerges as the predominant factor for the spatially consistent pattern (EOF1) that dominates the NCP and Eastern China. SIAI plays a secondary but substantial role, and North Atlantic variability (NAO and SST_NTA_) also exerts regulatory influences.

**Figure 6:**
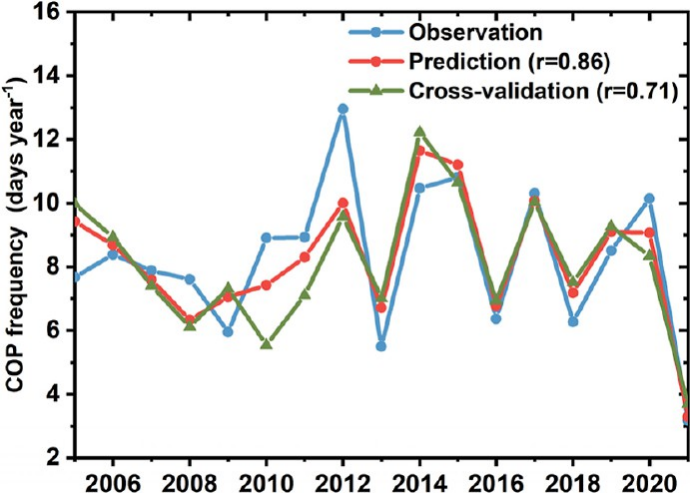
Multivariable regression modeling in NCP. Time series of annual COP frequency (days year^–1^) during May–July from 2005 to 2021 in NCP. Prediction using the MLR model and observations are represented in red and blue. The inferred COP by the 10-fold cross-validation method is denoted in green.

## 4 Summary

The roles of climate factors on O_3_ or PM_2.5_ pollution in China have been separately investigated, yet the understanding of the co-occurrence remains limited. Three major modes of interannual COP frequency in Eastern China are identified by EOF analysis, revealing a consistent spatial pattern, North–south dipole pattern, and high sensitivities in coastal regions of Eastern China. As shown in Figure 7, we attribute these leading modes to cooling SST in Western Pacific Ocean, Arctic SI loss near the Barents Sea, and North Atlantic tripole SST associated with NAO, respectively. Our findings provide new insights into the major modes of COP frequency in China and their underlying mechanisms, emphasizing the significant influence of preseasonal SST and SI anomalies.

**Figure 7:**
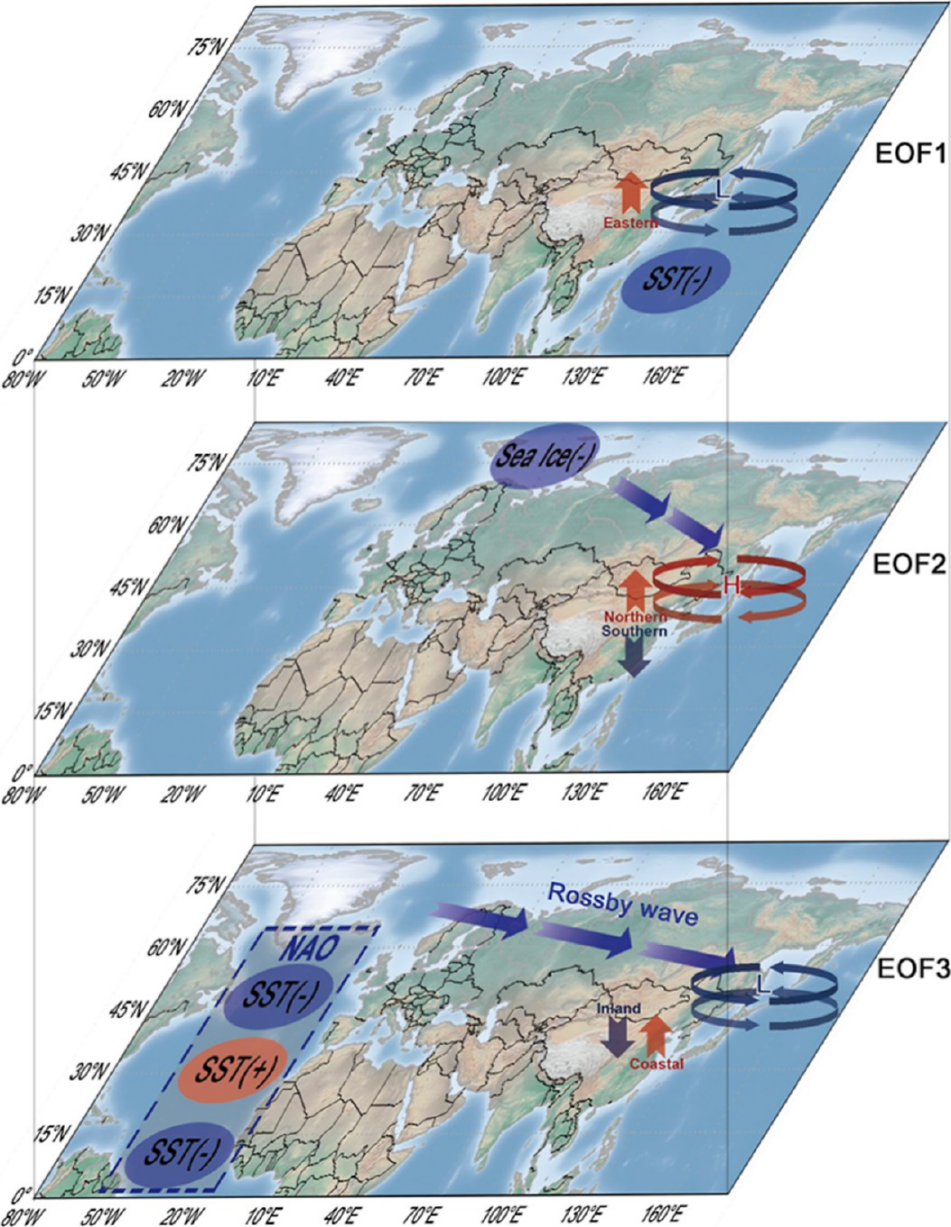
Schematic diagrams of the associated mechanisms. Conceptual scheme of modulation of leading modes of the COP frequency in Eastern China by preseasonal cooling SST in the Western Pacific, Arctic SI loss near the Barents Sea, and North Atlantic tripole SST anomalies associated with NAO.

The cooling SST in the Western Pacific Ocean is linked to weak WPSH, which promotes pollutant accumulation and reduces moisture transport to the NCP. The loss of Arctic SI near the Barents Sea triggers an atmospheric wave train, resulting in an anomalous anticyclonic circulation in Northeast China. It leads to a North–south dipole pattern with increased COP frequency in Northern China and reduced frequency in the YRD. The North Atlantic tripole SST anomalies associated with NAO induce a Rossby wave across the northern Eurasia continent with negative geopotential anomalies over Okhotsk Sea, contributing to increased air pollutants and insufficient moisture transport to the coastal regions of Eastern China. The development of a prediction model based on SST_wp_, SIAI, and NAO indices demonstrates the potential for forecasting COP frequency three months ahead. The model achieved significant correlation coefficients, particularly over the NCP. These findings could assist the Chinese government in implementing proactive measures to mitigate health risks associated with joint exposure.

Under a warming climate, SST in the Western Pacific Ocean is projected to increase by 2 °C due to the northward expansion of the subtropical gyre.^60,61^ This warming trend may improve air quality in Eastern China by reducing the likelihood of COP-favorable conditions. In contrast, the most significant projected Arctic SI loss is centered near Barents Sea,^62^ which could enhance COP frequency in Northern China. Furthermore, the NAO index is projected to continue fluctuating with a slight positive trend,^63^ potentially contributing further to increased COP occurrences. Previous modeling studies also suggest that climate change would increase PM_2.5_ and O_3_ concentration by 9 μg m^–3^ and 15.6 μg m^–3^ in NCP, respectively, by the middle of the century.^64,65^ While our study enhances our understanding of the climate influences on COP frequency, the role of anthropogenic emissions remains a critical factor. The interaction between emissions and climate variability warrants further investigation, particularly in the context of shifting emission trends and regulatory measures. Future research will aim to incorporate emission variations into the model to further enhance the predictive accuracy.

## Supplementary Material



## Notes

Figures of spatiotemporal distribution of COP, time series of 95th percentile thresholds for O_3_ and PM_2.5_ concentrations, SST and SI anomaly imposed in CESM simulation, spatiotemporal distribution of O_3_ and PM_2.5_ pollution events, correlations between the first three modes and SST, CESM-simulated responses to SST_wp_, regression of meteorological variables on PC2, CESM-simulated responses to SIAI, regression of meteorological variables on PC3 and NAO, wave activity flux associated with PC3, CESM-simulated responses to NAO, multivariable regression modeling in Eastern China and YRD, tables of AIC values for different combinations of predictors, and hierarchical partitioning analysis (PDF)
