# Supplementary material for: Foreseeable Co-occurring O3 and PM2.5 Pollution in Eastern China Driven by Climate Teleconnections
Source: ACS Environ Au. 2025 Oct 7;5(6):625–35. doi: 10.1021/acsenvironau.5c00164 (PMC12635941; doi:10.1021/acsenvironau.5c00164)
Supplement: Supplementary file 1 [file vg5c00164_si_001.pdf]

Supporting Materials for

Foreseeable co-occurring O_3 and $PM_{2.5}$ pollution in Eastern China driven by climate teleconnections

Xiaorui Zhang[1, 2], Meng Gao[1, 2, *], Gregory R. Carmichael[3]

1 Key Laboratory for Geographical Process Analysis and Simulation of Hubei Province, College of Urban and Environmental Sciences, Central China Normal University, Wuhan, China

2 Department of Geography, Hong Kong Baptist University, Hong Kong SAR 999077, China

3 John A. Paulson School of Engineering and Applied Sciences, Harvard University, Cambridge, MA 02138

4 Department of Chemical and Biochemical Engineering, The University of Iowa, Iowa City, IA 52242

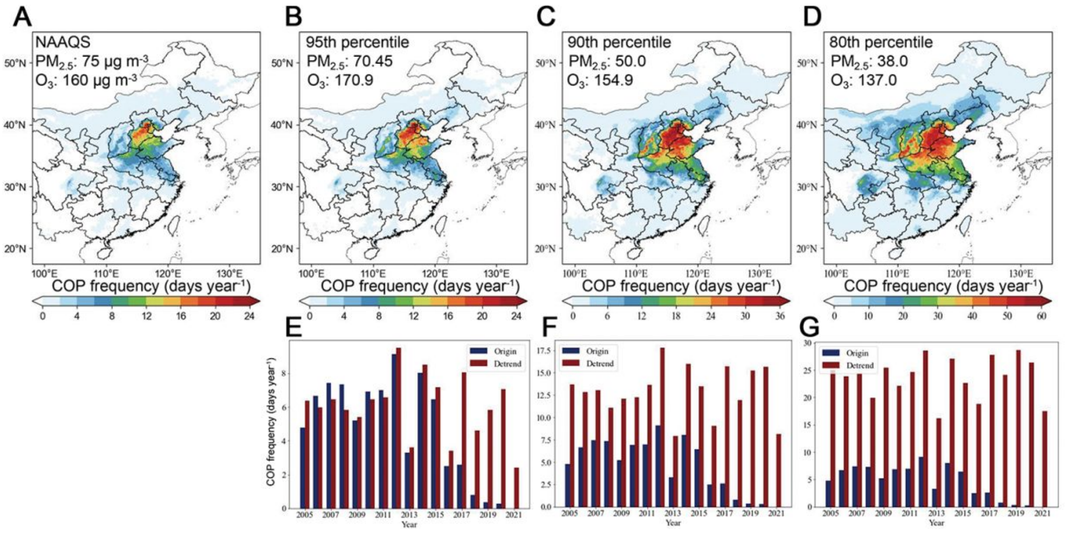

Fig. S1. Spatial distribution of averaged (A) co-occurring O_3 and $PM_{2.5}$ pollution (COP) frequency (days year^{-1}) defined by Grade II National Ambient Air Quality Standard of China (GB3095-2012), detrended COP frequency by (B) 95th percentile, (C) 90th percentile and (D) 80th percentile, and associated (E, F, G) annual variation of original (blue bars) and detrended (red bars) COP frequency (days year^{-1}) in NCP during May-July from 2005 to 2021.

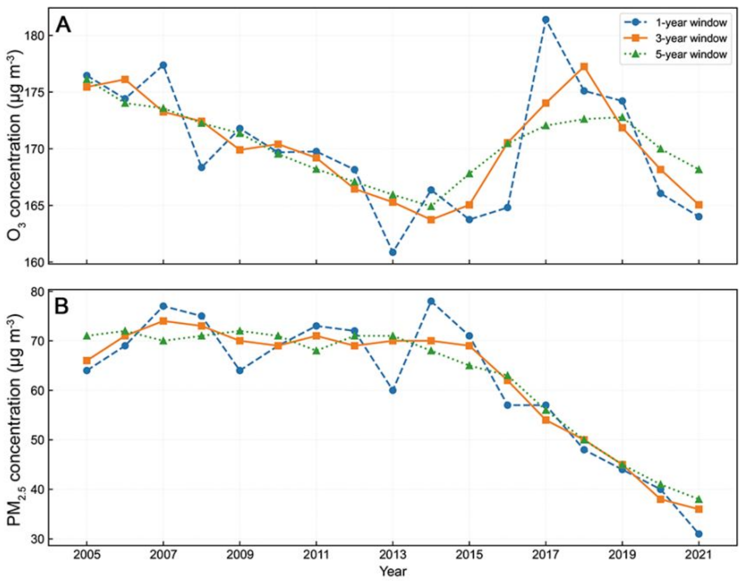

Fig. S2. Time series of 95th percentile thresholds for (A) O_3 and (B) $PM_{2.5}$ concentrations during 2005–2021, calculated using 1-year, 3-year, and 5-year sliding windows.

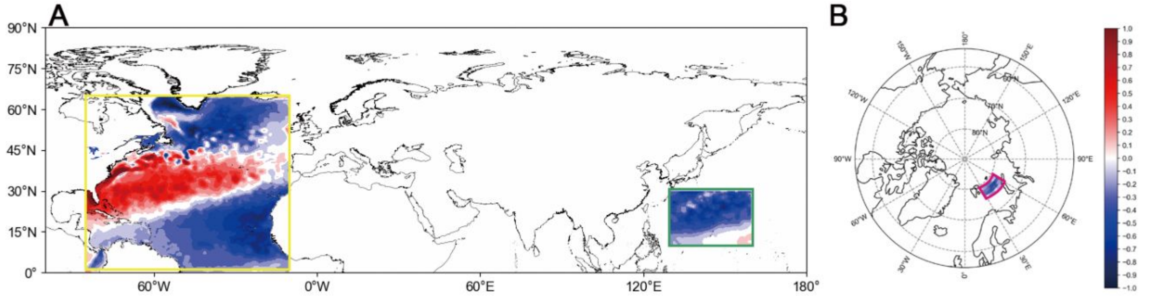

Fig. S3. Regression of (A) February-April averaged sea surface temperature (K) during 2005-2021 on SST_{wp} index in western Pacific, and NAO index in North Atlantic (The green and yellow box represent the region with SST anomaly imposed in $CESM_{wp}$ and $CESM_{NAO}$, respectively), and (B) SI (%) during April during 2005-2021 on SIAI index in Barents area (Pink box represent the region with SST anomaly imposed in $CESM_{wp}$).

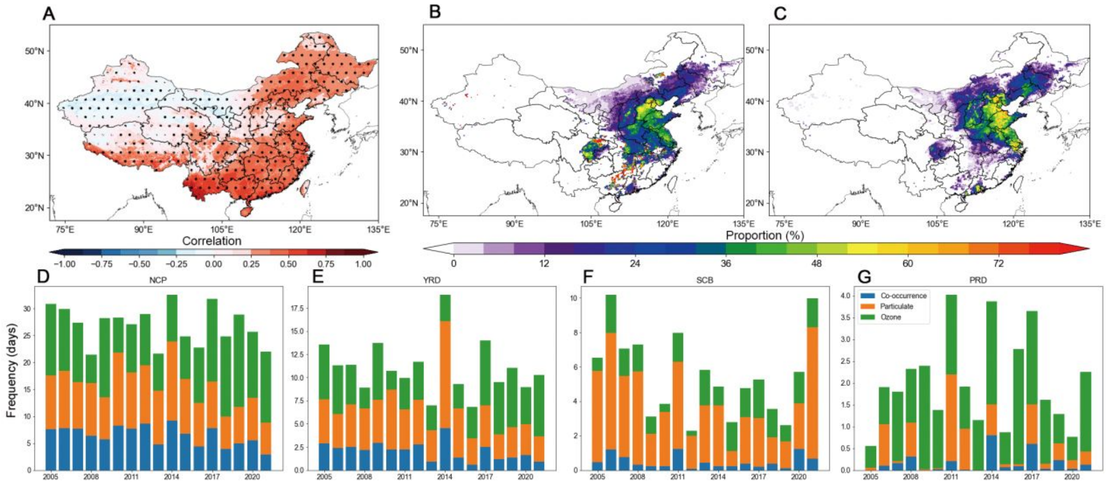

Fig. S4. (A) Correlation between daily O_3 and $PM_{2.5}$ concentration. Propotion of COP events among (B) O_3 pollution events, and (C) $PM_{2.5}$ pollution events. Interannual variations in the frequency (days) of COP events (blue bars), only $PM_{2.5}$ pollution events (orange bars) and only O_3 pollution events (green bars) in (D) NCP, (E) YRD, (F)

Sichuan Basin (SCB) and (G) Pearl River Delta (PRD) during May-July from 2005 to 2021.

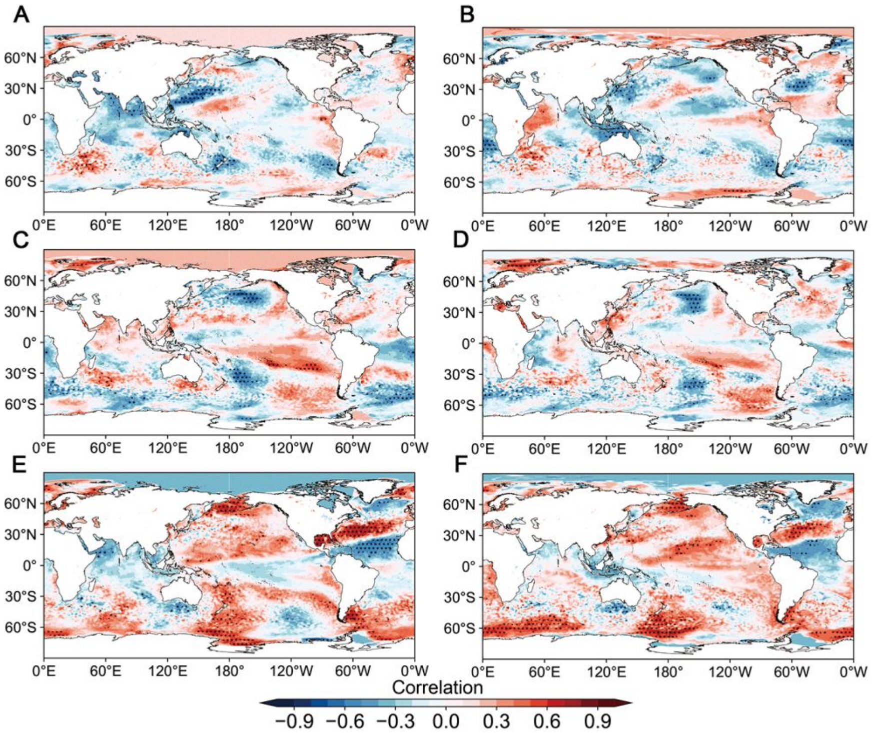

Fig. S5. Correlations between the first three modes and (A, C, E) SST during February-April, (B, D, F) during May-July from 2005 to 2021. Black dots denote areas with significant correlation ($P < 0.05$).

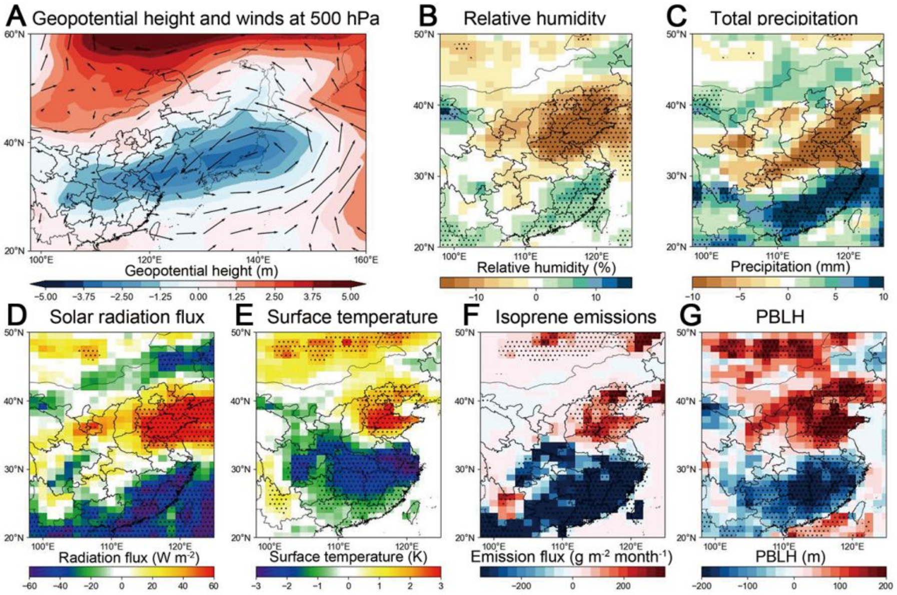

Fig. S6. CESM simulated response of (A) geopotential height (m) and winds (m s^{-1}) at 500 hPa, (B) relative humidity (%), (C) total precipitation (mm), (D) solar radiation flux (W m^{-2}), (E) surface temperature (K), (F) isoprene emissions flux (g m^{-2} $month^{-1}$) from Model of Emissions of Gases and Aerosols from Nature (MEGAN), and (G) planetary boundary layer height (PBLH, m) to SST_{wp}.

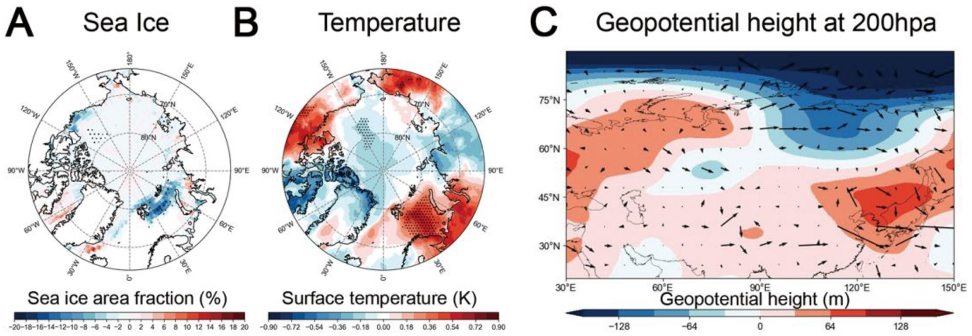

Fig. S7. Regression of (A) sea ice area fraction (%), (B) surface temperature (K), (C) geopotential height (m) and corresponding wave activity flux (vectors) at 200 hPa during May to July from 2005 to 2021 on PC2.

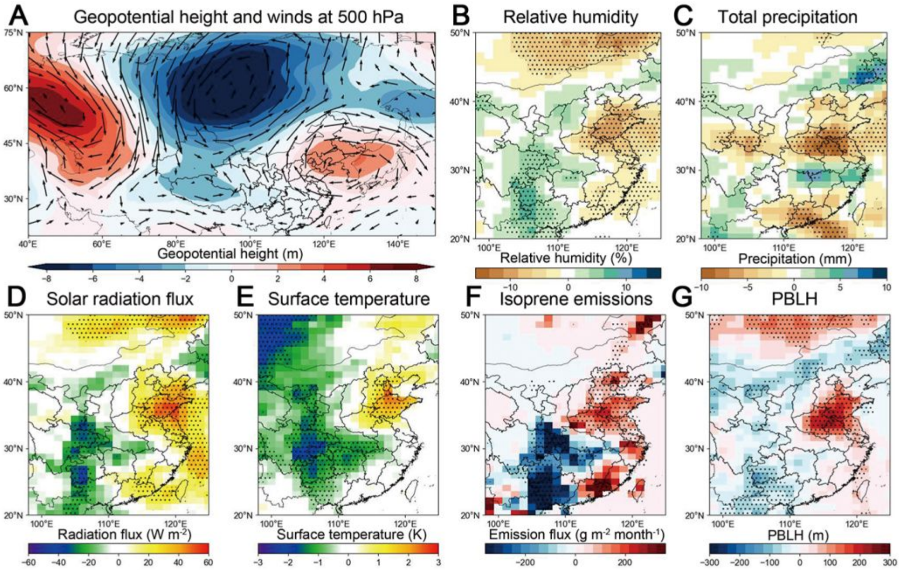

Fig. S8. CESM simulated response of (A) geopotential height (m) and winds (m s^{-1}) at 500 hPa, (B) relative humidity (%), (C) total precipitation (mm), (D) solar radiation flux (W m^{-2}), (E) surface temperature (K), (F) isoprene emissions flux (g m^{-2} $month^{-1}$) from MEGAN, and (G) PBLH (m) to SIAI.

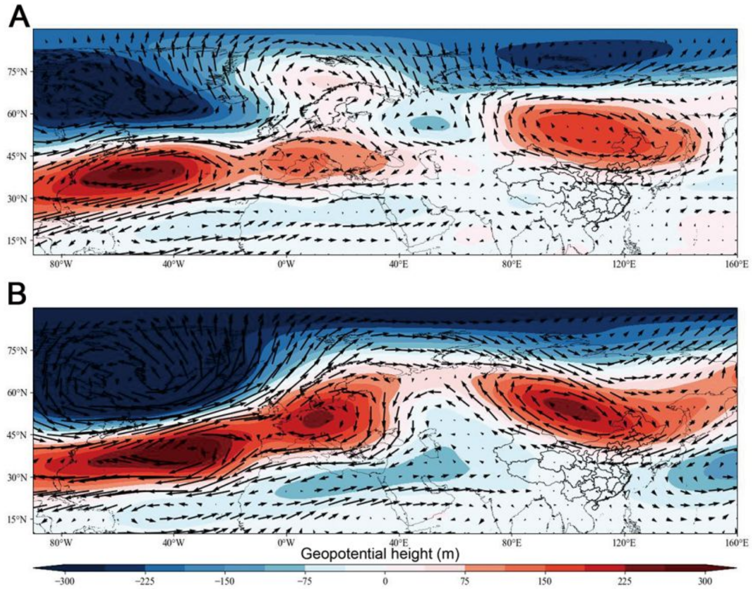

Fig. S9. Regression of geopotential height (m) and winds (m s^{-1}) at 500 hPa during February to April from 2005 to 2021 on (A) PC3 and (B) NAO.

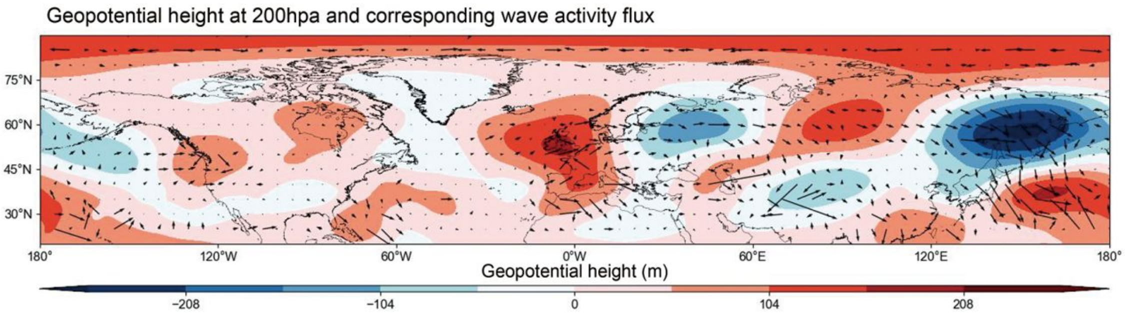

Fig. S10. Regression of geopotential height (m) and corresponding wave activity flux (vectors) at 200 hPa during May to July from 2005 to 2021 on PC3.

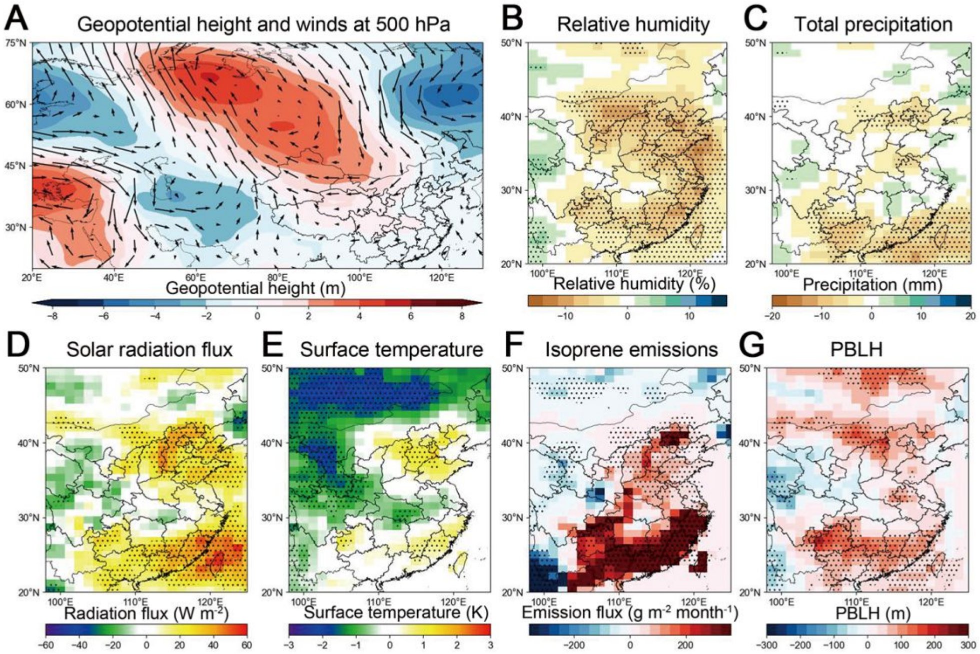

Fig. S11. CESM simulated response of (A) geopotential height (m) and winds (m s^{-1}) at 500 hPa, (B) relative humidity (%), (C) total precipitation (mm), (D) solar radiation flux

(W m^{-2}), (E) surface temperature (K), (F) isoprene emissions flux (g m^{-2} month^{-1}) from MEGAN, and (G) PBLH (m) to NAO.

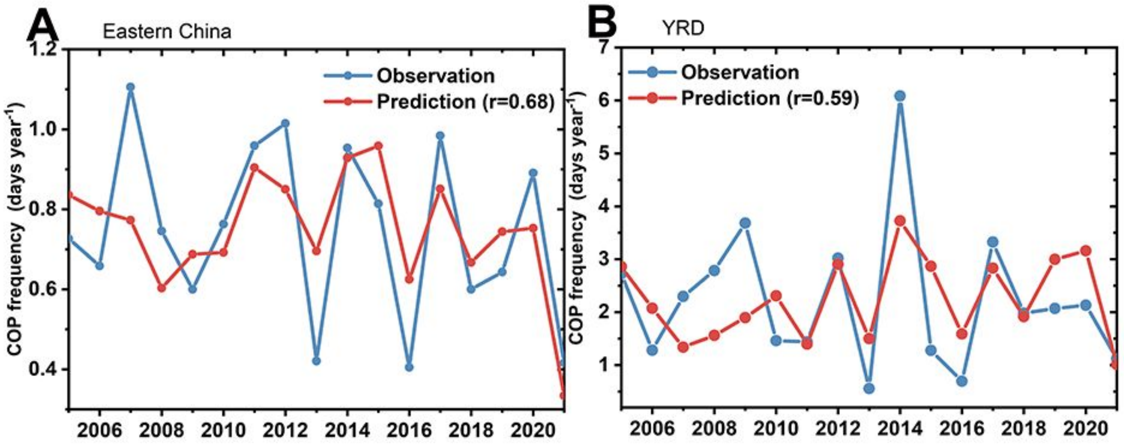

Fig. S12. Time series of annual COP frequency (days year^{-1}) during May-July from 2005 to 2021 in (A) Eastern China and (B) YRD. Prediction using MLR model and observations are represented in red and blue.

Table S1. AIC values for different combinations of predictors.

Combinations of predictors	AIC values
SST_{wp}	68.51
SIAI	75.57
NAO	79.48
SST_{NTA}	80.11
SST_{wp}+SIAI	62.95
SST_{wp}+NAO	66.83
SST_{wp}+SST_{NTA}	67.31
SIAI+NAO	74.02
SIAI+SST_{NTA}	74.93
SST_{wp}+SIAI+NAO	62.54
SST_{wp}+NAO+SST_{NTA}	65.61
SST_{wp}+SIAI+SST_{NTA}	62.86
SIAI+NAO+SST_{NTA}	73.71
SST_{wp}+SIAI+NAO+SST_{NTA}	62.36

Table S2. Hierarchical partitioning analysis.

Regions	Total explained variance	Relative contribution

		SST_{wp}	SIAI	NAO	SST_{NTA}
NCP	0.61	61.1%	20.9%	6.1%	11.9%
Eastern China	0.46	64.8%	21.7%	5.9%	7.5%
YRD	0.26	36.8%	29.6%	15.4%	18.2%
